# Optimizing drug combinations to resurrect the potency of failed antibody therapy against emerging COVID-19 variants using IDentif.AI

**DOI:** 10.3389/fdgth.2026.1744623

**Published:** 2026-06-02

**Authors:** Peter Wang, Kui You, Zi Wei Chia, Lissa Hooi, Li Ming Chong, De Hoe Chye, Angelina Moh, Ze Yong Lee, Ethan Lim, Alrick Zi Xin Kok, Stephen Chua, Isaiah Zhuang, Ella Chang, Boon Huan Tan, Gladys Gek Yen Tan, Shawn Vasoo, Conrad E. Z. Chan, Edward Kai-Hua Chow, Dean Ho

**Affiliations:** 1 Department of Biomedical Engineering, College of Design and Engineering, National University of Singapore, Singapore, Singapore; 2 Institute for Digital Medicine (WisDM), Yong Loo Lin School of Medicine, National University of Singapore, Singapore, Singapore; 3 The N.1 Institute for Health (N.1), National University of Singapore, Singapore, Singapore; 4 National Centre for Infectious Diseases (NCID), Singapore, Singapore; 5 Cancer Science Institute of Singapore, National University of Singapore, Singapore, Singapore; 6 Defense Medical and Environmental Research Institute, DSO National Laboratories, Singapore, Singapore; 7 Department of Pharmacology, Yong Loo Lin School of Medicine, National University of Singapore, Singapore, Singapore; 8 Repiratory and Infectious Disease Program, Lee Kong Chian School of Medicine, Nanyang Technological University, Singapore, Singapore; 9 The Bia-Echo Asia Centre for Reproductive Longevity and Equality (ACRLE), National University of Singapore, Singapore, Singapore

**Keywords:** combination therapy, COVID-19, digital medicine, infectious disease, optimization

## Abstract

**Introduction:**

Since the outbreak of the COVID-19 pandemic, extensive efforts including vaccine and drug development have been accentuated to address the emergence of SARS-CoV-2 variants. The rising variants and subvariants made early discoveries in effective treatment strategies less relevant and potent. For instance, multiple therapeutics like Evusheld are no longer effective against current variants and therefore have their emergency use authorizations (EUA), a regulatory mechanism allowing emergency use of medical products, revoked until further notice. Similarly, sotrovimab (STV), a human neutralizing monoclonal antibody (MAB), received EUA in May 2021 and was later granted authorization for COVID-19 treatment in Singapore. However, mutations presented in XBB, a subvariant of Omicron, substantially reduced the potency of STV at the currently approved dose.

**Methods:**

To retain STV as a part of the treatment strategy for COVID-19 infected patients, it is important to carefully design and prioritize drugs that may further enhance the potency of STV when paired in combinations. In this study, IDentif.AI, an artificial intelligence (AI)-derived drug combination optimization platform, was harnessed to rapidly pinpoint STV-based combinations that may substantially improve the efficacy and potency of STV against XBB, which was predominantly spreading worldwide in 2023.

**Results:**

IDentif.AI-pinpointed STV/EIDD-1931 and STV/GS-441524 combinations were able to interact synergistically to enhance efficacy against XBB and importantly, substantially reduce STV's in vitro EC50 (half maximal effective concentration) by up to 7-fold.

**Discussion:**

This optimization platform represents a strategic approach to rapidly repurpose existing or previously failed therapeutics and subsequently restore their efficacy against a disease indication by pairing them with the correct drugs in combinations.

## 1 Introduction

Since its emergence in late 2019, the COVID-19 pandemic has presented an unprecedented challenge to global public health systems. As the SARS-CoV-2 virus has spread and evolved, the emergence of new variants has necessitated continual adaptations in treatment approaches. Since the initial outbreak, numerous SARS-CoV-2 variants have been identified, with some classified as variants of concern (VOCs) due to their increased transmissibility, severity, or ability to evade immune responses. Notable VOCs include Alpha, Beta, Gamma, Delta, and Omicron. Each new variant has brought distinct challenges, often reducing the efficacy of existing treatments and vaccines, thereby compelling the healthcare community to reassess and modify treatment protocols. Notably, in early 2023, due to declining efficacy against emerging variants, United States (US) Food and Drug Administration (FDA) revoked the EUA of Evulsheld, a monoclonal antibody (MAB) combination, until further notice (1). Emerging variants have influenced treatment strategies in several key areas. These include antiviral therapies, where the effectiveness of early treatments against emerging variants has been examined; monoclonal antibody treatments, which have required adaptations in antibody cocktails to maintain efficacy; and immunomodulatory approaches, involving changes in the use of corticosteroids and other immuno-modulating drugs. Furthermore, advancements in healthcare technologies including artificial intelligence (AI)-powered digital platforms have transformed methodologies for designing therapeutics and formulating treatment strategies (2–5). Stochastic search algorithm, higher-order drug combination optimization, and other statistical models have also been harnessed to design synergistic drug combinations for various disease indications (6–9). Moreover, integrating generative AI for *de novo* drug design and pairing AI with existing high throughput drug screening technologies have also led to a helpful framework and blueprint for the future of both drug development and pandemic readiness (5).

Emerging SARS-CoV-2 variants have led to the declining potency of multiple drugs including sotrovimab (STV), a MAB granted EUA for COVID-19 treatment (10). Some drugs like STV that have previously obtained EUA are either no longer effective or not authorized for use. Aside from developing targeted therapies, restoring the efficacy of drugs that are no longer active against variants through combinatorial designs may be a strategic approach for COVID-19 treatment as well as for other disease indications. In this study, to resurrect and repurpose these drugs, IDentif.AI was harnessed to pinpoint combinatorial designs that may enhance the potency of STV for COVID-19 treatment. This platform solely relies on prospectively generated, small datasets and correlates biological response to drug combinations (e.g., %Inhibition) via an AI-discovered second order quadratic series, which interrogates the drug-drug interaction space (11–18). The first iteration of IDentif.AI involved using neural networks to determine the relationship between drug interventions and cellular responses, and the modeling of the relationship can be approximated by a second order quadratic series (11). This small data-driven approach has been validated in multiple *in vitro, in vivo,* and human studies that investigated drug combinations for infectious diseases (e.g., *Enterobacteriaceae*), heart disorder, and oncology (12–16, 19–26). Importantly, IDentif.AI does not use pre-existing drug information, big data, or *in silico* modeling to optimize drug combinations (13, 16, 22, 26). This work differs substantially from previous studies, which primarily focused on optimizing effective drug combinations against infectious diseases including COVID-19 and *Enterobacteriaceae*. However, in this study, the IDentif.AI platform is repositioned to design combinations revolving around failed therapies such as STV to resurrect their potency against emerging SARS-CoV-2 variants and potentially other indications. The potency of FDA EUA-revoked drugs can potentially be resurrected with the right combinations as pinpointed by IDentif.AI. Therefore, this study aims to harness the IDnetif.AI platform to optimize and prioritize STV-based drug combinations that may potentially restore STV potency against SARS-CoV-2 Omicron XBB subvariants.

**Figure 1:**
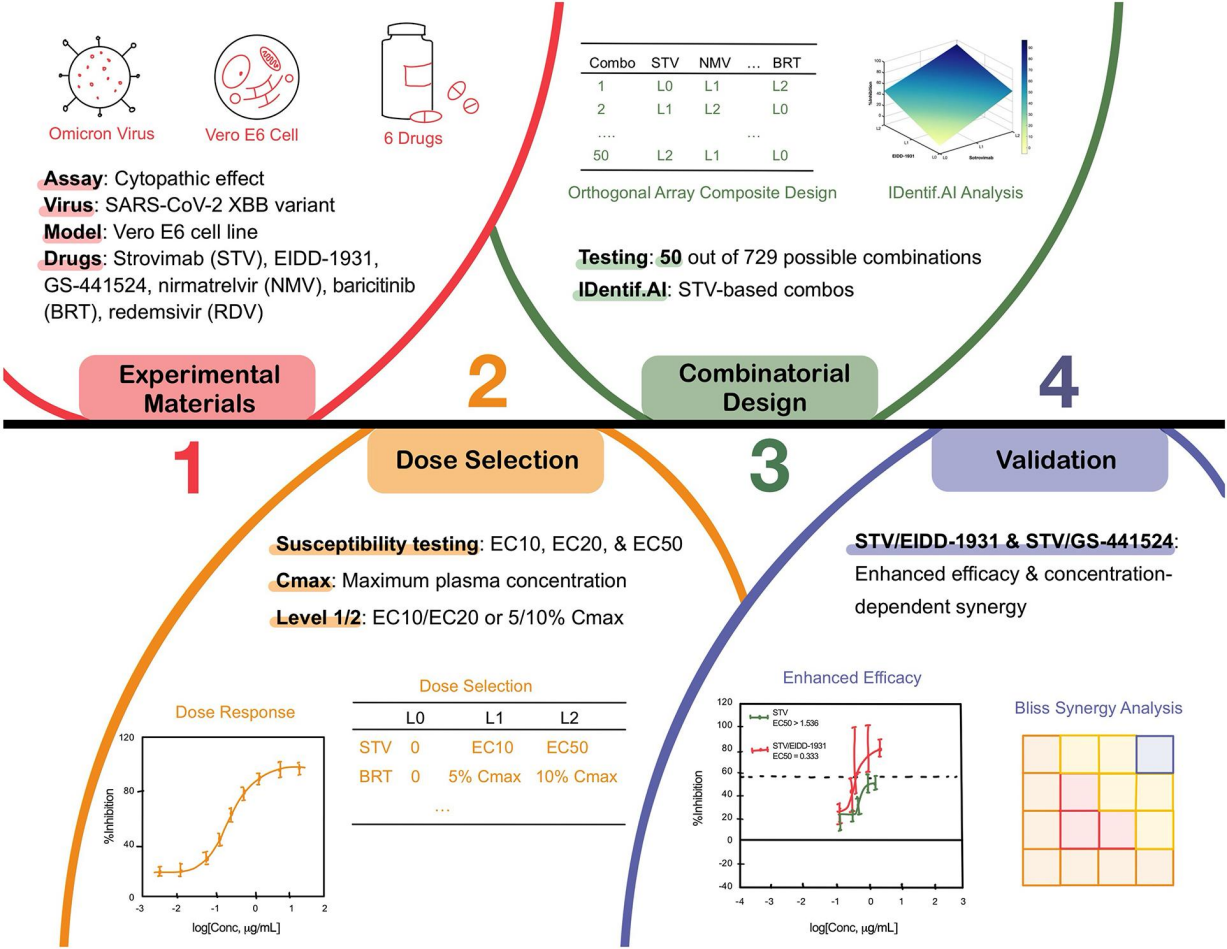
IDentif.AI workflow rapidly pinpoints drug combinations that may restore the potency of STV against XBB. The IDentif.AI workflow is consisted of four major stages: (1) The initial stage of the workflow involves active engagement with clinical teams including infectious diseases experts to determine an initial pool of clinically relevant drugs and the experimental design including relevant assays. (2) Afterwards, the selected drug candidates are assessed individually to derive their dose response curves, which provide important information, such as the EC_10_, EC_20_, and EC_50_ of the drugs. Aside from these values, the pharmacological data (e.g., C_max_) derived from clinical studies are also incorporated to the selection of L1 and L2 drug concentrations. (3) IDentif.AI analysis interrogates the drug interaction space (729 combinations) using the experimentally derived %Inhibition values of 50 combinations, and the platform rapidly prioritizes actionable STV-based combinations. (4) STV/EIDD-1931 and STV/GS-441524 combinations were prioritized and comprehensively validated and assessed via synergy and dose response analyses.

To resurrect the potency of STV against the SARS-CoV-2 Omicron XBB subvariant, IDentif.AI interrogated the interaction space of STV with five additional drugs: nirmatrelvir (NMV), remdesivir (RDV), GS-441524 (parent nucleoside of remdesivir), baricitinib (BRT), and EIDD-1931 (molnupiravir). The platform prioritized two STV-based 2-drug combinations—STV/EIDD-1931 and STV/GS-441524—that were able to reduce the *in vitro* EC_50_ (half maximal effective concentration) of STV by up to 7-fold. Additional interaction analysis of both combinations determined that they exhibited dose-dependent synergy. Importantly, no apparent cytotoxicity was observed across all tested concentrations. In sum, this study presented an alternative strategy to restore the efficacy of drugs that were no longer active against emerging variants/subvariants and to further diversify treatment options for COVID-19 patients (Figure 1). The use of drug combinations with heterogeneous mechanisms of action may also act to reduce the emergence of drug resistance. Additionally, this platform may also be repositioned to address other challenges for pandemic readiness, such as pinpointing combinations for other pathogens (e.g., antimicrobial resistance, AMR) that currently have no effective treatment options.

## 2 Materials and methods

### 2.1 Experimental model

All experimental work involving live SARS-CoV-2 XBB virus was performed in a biosafety level-2 (BSL-2) laboratory. The XBB subvariant of SARS-CoV-2 (Omicron) was a local isolate cultured from a nasopharyngeal swab. Vero E6 cells (African green monkey kidney epithelial cell line) overexpressing ACE2 (angiotensin-converting enzyme 2) and TMPRSS2 (transmembrane serine protease 2) served as the infection model for this experiment. Each drug treatment—sotrovimab (STV), nirmatrelvir (NMV), remdesivir (RDV), GS-441524, baricitinib (BRT), and EIDD-1931—was prepared in wells containing culture media. Vero E6-ACE2-TMPRSS2 cells (2 × 10^4^ cells/well) were incubated with SARS-CoV-2 live virus at 178 TCID_50_ (median tissue culture infectious doses; approximately 125 infectious particles; multiplicity of infection (MOI) = 0.00625) for 1 h at 37 °C in a 96-well plate. After thorough assay optimization, these infection conditions were selected to achieve measurable viral infection. The cells were further incubated for 72 h at 37 °C with 5% CO_2_ to allow for virus-induced cytopathic effect (CPE) to develop, which was used to calculate %Inhibition. CPE was measured using Viral ToxGlo Assay (Promega, G8941) as a readout of cell viability. To determine the %Inhibition of each monotherapy or combination, the measured CPE inhibition was normalized to positive and negative controls, which are cells treated with vehicle (DMSO; dimethyl sulfoxide) only and ones exposed to virus and DMSO, respectively. In contrast, %Cytotoxicity was derived from the same plate layout for monotherapies and drug combinations but without the addition of live virus to the plates.

### 2.2 Selection of clinically achievable drug concentrations

The %Inhibition and %Cytotoxicity of monotherapies of the selected drugs were derived individually in 12 different concentrations via 2-fold serial dilution dose response experiments (Supplementary Figure S1). Subsequently, the prospectively acquired monotherapy data were analyzed in GraphPad Prism 9 (GraphPad Inc.) and the EC_10_ (10% effective concentration), EC_20_ (20% effective concentration), and EC_50_ values of each drug were extracted from the dose response curves. The IDentif.AI analysis assessed three concentration levels: Level 0 (L0), Level 1 (L1), and Level 2 (L2), which correspond to the absence of drug and two clinically achievable drug concentrations. To optimize actionable drug combinations, clinically relevant and achievable drug concentrations derived from reported C_max_ (maximum serum/plasma concentration) values in FDA documents and clinical trials were incorporated into the IDentif.AI analysis. Generally, 10% of C_max_ is considered an achievable drug concentration at targeted sites in human. Therefore, L1 and L2 drug concentrations were selected based on the lower of EC_10_/EC_20_ or 5%/10% of C_max_, ensuring concentrations assessed were clinically achievable (Table 1).

**Table 1:** Monotherapy assessment from dose response curves and pharmacological data were used to determine the L1 and L2 concentrations for IDentif.AI analysis.

Drug	EC_50_ (µM)	Maximum plasma concentration (C_max_)		IDentif.AI analysis	
		Conc. (µM)	Dosing scheme	Level 1 (µM)	Level 2 (µM)
NMV	9.870	1.762 (27)	A single oral dose of 250 mg suspension formulation	0.0881	0.176
RDV	2.969	3.699 (28)	Multiple IV doses of 100 mg VEKLURY	0.185	0.370
GS-441524	0.493	0.498 (28)	Multiple IV doses of 100 mg VEKLURY	0.0249	0.0498
BRT	>10	0.14 (29)	NA	0.007	0.014
STV^a^	2.449	170.1 (30)	A single IV dose of 500 mg sotrovimab	0.315	0.768
EIDD-1931	0.886	8.989 (31)	Multiple oral doses of 800 mg LAGEVRIO capsule every 12 h	0.251	0.448

NMV, nirmatrelvir; RDV, remdesivir; BRT, baricitinib; and STV, sotrovimab.

a The unit of STV concentration is in µg/mL.

### 2.3 IDentif.AI: optimization of STV-based drug combinations

In this study, a curated set of 50 drug combinations consisting of three concentrations levels—L0, L1, L2—was designed according to a 6-drug resolution VI orthogonal array composite design (OACD) (Supplementary Table S1). Specifically, L1/L2 concentrations were selected based on dose response parameters or pharmacokinetic C_max_ values, ensuring drug-drug interactions are detected in clinically achievable concentrations. This resolution VI OACD was generated by combining 32 two-level fractional factorials and 18 three-level orthogonal arrays (32). More importantly, these 50 drug combinations represented the minimum number of data required for IDentif.AI to efficiently screen the inhibitory effects of each drug through their linear, bilinear (drug-drug interactions), and quadratic coefficients. To further elaborate, all 50 OACD combinations were added to Vero E6-ACE2-TMPRSS2 cells infected by SARS-CoV-2 live virus, and the %Inhibition efficacy of all combinations were experimentally measured. Subsequently, IDentif.AI correlated the input drug interventions with their corresponding measured %Inhibition data via an AI-discovered biological relationship (11), which resulted in an actionable second order quadratic series that can be used to interrogate the entire drug-drug interaction space comprised of all 729 possible combinations (6 drugs at three concentrations levels: 3^6^ = 729) (*N* = 3) (MATLAB R2020b, MathWorks Inc.) (Supplementary Tables S1 and S2). Box-Cox transformation was applied to the %Inhibition dataset to determine an appropriate transformation that may improve the residual distribution and importantly, the goodness-of-fit of IDentif.AI analysis (e.g., adjusted R^2^). Lastly, utilizing the second order quadratic series, IDentif.AI was able to provide a ranked list of all 729 combinations along with their corresponding predicted %Inhibitions. Thus, IDentif.AI, a small-data driven approach, was able to rapidly screen the drug-drug interaction space using only a set of 50 prospectively screened drug combinations. Furthermore, a series of residual-based outlier analyses were performed to detect potential outliers in the %Inhibition dataset (Supplementary Figure S3). Specifically, the plot of residuals vs. fitted values was used to assess linearity and homoscedasticity of residuals, where random distribution of residuals around zero indicated acceptable model fit. The Cook's distance plot was used to identify influential outliers among the fitted data. Lastly, the normal probability plot of residuals and histogram of residuals both evaluated the normality of residual distribution, with approximate normality indicating acceptable model assumptions (linear regression).

### 2.4 Synergy analysis

The %Inhibition and %Cytotoxicity of the two IDentif.AI-pinpointed STV-based combinations were further assessed via a 7 × 7 checkerboard assay (*N* = 3). The highest concentration of STV evaluated in the checkerboard assay was set at 2*x* the original L2 concentration listed in Table 1 due to the high potency of STV at the L2 concentration (Supplementary Figure S2). Limiting the highest STV concentration to 2*x* of L2 enabled insightful assessment of drug-drug interactions in STV-based combinations while avoiding overrepresentative effects from STV. In contrast, EIDD-1931 and GS-441524 exhibited substantially lower efficacy at their respective L2 concentrations and therefore, their highest concentrations in the checkerboard assay were set at 4*x* of their L2 concentrations to allow broader evaluation of drug-drug interactions. 2-fold serial dilution was carried out for each drug in the checkerboard assay. The tested concentration ranges for STV, EIDD-1931, and GS-441524 were 0–1.536 µg/mL, 0–1.792 µM, and 0–0.199 µM, respectively. The experimentally derived %Inhibition data of both combinations at different dose ratios were used to generate the interaction maps (GraphPad Prism 9, GraphPad Inc.). Subsequently, individual %Inhibition replicates (*N* = 3), instead of averaged values, were uploaded to SynergyFinder + online tool to assess the drug-drug interactions of the two combinations using Bliss independence, zero interaction potency (ZIP), Loewe, and the highest single agent (HSA) models (12, 16, 19, 22, 33). Similarly, the final synergy scores corresponding to each dose ratio on the 7 × 7 checkerboard were also determined from all three replicates (*N* = 3). The synergy scores were downloaded and inputted into GraphPad Prism 9 to generate synergy maps (GraphPad Inc.). The same procedure was followed for the %Cytotoxicity data of the two STV-based combinations. Synergy scores of < −10, between −10 and 10, and > 10 indicate antagonistic, additive, and synergistic interactions, respectively. The statistical significance of the synergy scores was determined using one-sample student *t*-test. Sum of squares *F*-test was used to determine statistical significance between STV and STV-based combinations in the dose response curves.

### 2.5 Statistical analysis

All experiments were performed in triplicates (*N* = 3). Data are presented in mean ± propagated SD (12, 13, 16, 34). Z'-factor was calculated for each set of experiments to determine the assay quality based on positive and negative controls (35). Shapiro–Wilk normality test was performed to examine the distribution of experimental data. The statistical significance of Bliss synergy scores and dose response curves was determined via one-sample *t*-test and sum of squares *F*-test, respectively (GraphPad Inc.). IDentif.AI analysis was performed using MATLAB's stepwise regression analysis (MATLAB 2020R; MathWorks Inc.), and the relevant statistics were obtained using the sum of squares *F*-test.

## 3 Results

### 3.1 Monotherapy assessment of drug candidates

To ensure clinical feasibility and relevance of the findings in this study, the initial pool of drugs was selected by consulting with infectious disease clinicians at the National Centre for Infectious Diseases (NCID), Singapore (Figure 1). Additionally, literature search also helped identify drug candidates that made the final list: sotrovimab (STV) (36), nirmatrelvir (NMV) (13), remdesivir (RDV) (22), GS-441524 (active metabolite of GS-621763) (37, 38), baricitinib (BRT) (13, 22), and EIDD-1931 (molnupiravir) (13, 22). Notably, all of the selected drugs have previously obtained FDA's EUA for COVID-19 treatment (39–41), except for GS-441524, which may potentially be an orally available alternative to RDV (37, 38). The selected drugs exhibit diverse mechanisms of action, which may prevent drug resistance. For example, STV neutralizes the spike protein of SARS-CoV-2 after binding to it and molnupiravir induces RNA mutations that impair viral replication. Pairing these drugs with STV may potentially restore the potency of the antibody therapy.

The %Inhibition efficacy of each of the selected drugs was determined by co-incubation with Vero E6-ACE2-TMRPSS2 cells infected with live SARS-CoV-2 Omicron XBB subvariant and virus-induced CPE was subsequently measured with a cell viability assay (*N* = 3). Uninfected cells were exposed to each drug at the same concentrations to derive the dose response curves for %Cytotoxicity. The monotherapy assessment of each drug was performed via dose response analysis (Supplementary Figure S1 and Data S1). This set of experiments had a Z'-factor of 0.58, indicating good assay quality. Of all drugs, BRT had insufficient efficacy against XBB even at the highest tested concentration. Furthermore, no apparent cytotoxicity was observed in the tested concentration ranges for the selected drugs. However, EIDD-1931 at 10 µM (the highest tested concentration) exhibited substantial cytotoxicity (> 15%), which resulted in a slight decline in %Inhibition at the same concentration (Supplementary Figure S1). As a result, the data point at 10 µM was removed from the dose response analysis when determining EC_50_ and relevant values for EIDD-1931. All other data points were included in the dose response analysis.

### 3.2 Selection of L1/L2 drug concentrations for IDentif.AI analysis

To optimize and design effective drug combinations, IDentif.AI assesses two clinically relevant concentration levels: L1 and L2. These two concentration levels were selected based on one of the two methods: (1) EC_10_ and EC_20_ derived from dose response curves and (2) 5% and 10% of C_max_ (maximum serum concentration) values determined from FDA documents and clinical trials. The L2 concentration was limited to EC_20_ ensuring that no single drug is overrepresented in a combination. Additionally, considering the C_max_ of a drug ensures that IDentif.AI results are clinically relevant and achievable. The L1 and L2 concentrations and other relevant data are summarized in Table 1. The concentration levels for most drugs were selected based on 5% and 10% of C_max_ as their EC_20_ values exceeded the clinically relevant concentration (10% of C_max_). In contrast, the L1 and L2 concentrations of STV and EIDD-1931 were selected based on EC_10_ and EC_20_ as their 10% of C_max_ were substantially higher than their EC_50_ values. This selection approach ensured that all L1/L2 concentrations were clinically achievable.

### 3.3 IDentif.AI-prioritized STV-based drug combinations

A set of 50 drug combinations according to a resolution VI orthogonal OACD was co-incubated with XBB infected Vero E6-ACE2-TMRPSS2 cells. The %Inhibition data of the combinations were experimentally determined via virus-induced CPE inhibition effects (*N* = 3) (Supplementary Figure S2), which resulted in a Z'-factor of 0.05 (doable assay) (35). No apparent cytotoxicity was observed across all 50 OACD combinations (Supplementary Data S1). The selected L1 and L2 concentrations summarized in Table 1 were also experimentally validated. The monotherapies at L1 and L2 concentrations were broadly ineffective, except STV, which exhibited more potency (> 20%Inhibition) than expected against XBB at the L2 concentration (Supplementary Figure S2). Subsequently, IDentif.AI correlated the input OACD combinations and L1/L2 monotherapies with their corresponding measured %Inhibition data via a second order quadratic series, which was used to interrogate the parameter space consisting of 729 combinations (6 drugs at three concentration levels; 3^6^ = 729) (Supplementary Table S2). The analysis resulted in an adjusted R^2^ of 0.71, indicated that the IDentif.AI model explained a substantial proportion of variability between the input drug interventions and corresponding measured %Inhibition data (Supplementary Table S2). No transformation was applied to the %Inhibition dataset prior to IDentif.AI analysis. Residual-based outlier analysis did not detect any outliers, and all data were included in the IDentif.AI analysis (Supplementary Figure S3).

**Figure 2:**
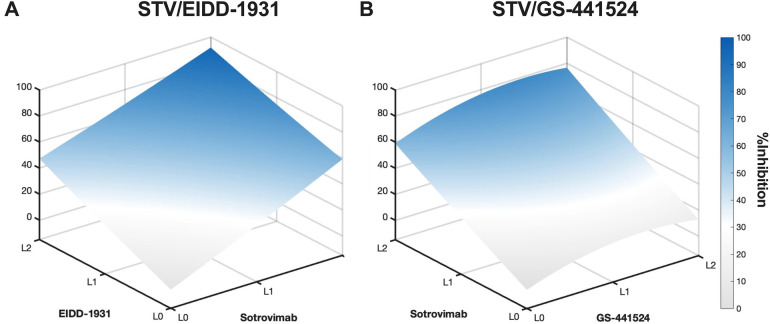
IDentif.AI-predicted interactions of STV-based combinations. **(A)** The interaction surface of STV/EIDD-1931 suggests that the combination is mostly driven by mild and additive interactions when drug concentrations achieve L2. **(B)** The interaction between STV and GS-441524 is mostly driven by STV. However, the interaction surface suggests that mild interaction may be achieved at L2 concentrations.

The IDentif.AI-estimated second order quadratic series (Supplementary Table S2) provided a ranked list of 729 combinations along with their predicted %Inhibition efficacy (Supplementary Data S2). Notably, this study aims to determine drugs that may enhance the potency of STV when paired in combinations. Therefore, efficacious STV-based 2-drug combinations were prioritized (Supplementary Table S3). The top ranked 2-drug combination consists of STV and EIDD-1931. An additional STV-based combination is STV in combination with GS-441524, which is the plasma metabolite of oral prodrug GS-621763/VV116 (42, 43). The prodrugs of EIDD-1931 (molnupiravir) and GS-441524 are orally available and may address the limitations of intravenous delivery including therapeutic compliance for non-hospitalized patients (43–45). As a result, RDV, which requires intravenous injection, in combination with STV was not prioritized (Supplementary Table S3). Furthermore, IDentif.AI analysis also predicted the drug-drug interactions of the combinations (Figure 2). The platform predicted that the interactions between STV and EIDD-1931 may be mostly driven by additive effects when both drugs achieve L2 concentrations (Figure 2A). In contrast, though the interaction of STV/GS-441524 is mostly driven by STV, the combination may exhibit mild synergistic interactions at the L2 concentrations (Figure 2B). These two combinations were further assessed in the subsequent validation experiment.

Moreover, these combinations may also serve as the backbone of combinatorial designs that further enhance the efficacy of STV against current and future emerging variants (Supplementary Tables S3 and S4). A few 3-drug and 4-drug combinations consisting of STV, EIDD-1931, and GS-441524 from the validation of 50 OACD combinations demonstrated high %Inhibition efficacy against XBB variant. For example, 4-drug combinations—STV/EIDD-1931/GS-441524/NMV (combination 22) and STV/EIDD-1931/GS-441524/RDV (combination 23)—were able to achieve near 100%Inhibition against the virus (Supplementary Data S1 and Tables S3 and S4). Another combination that pairs STV/EIDD-1931 with RDV (combination 43) was able to achieve 87.6 ± 21.7%Inhibition (Supplementary Data 1). Pairing additional drugs with STV/EIDD-1931 and STV/GS-441524 resulted in promising %Inhibition against the virus. Therefore, these two 2-drug combinations were prioritized for further analysis to determine their ability to restore the efficacy of STV against XBB.

### 3.4 Synergy analysis for STV-based drug combinations

**Figure 3:**
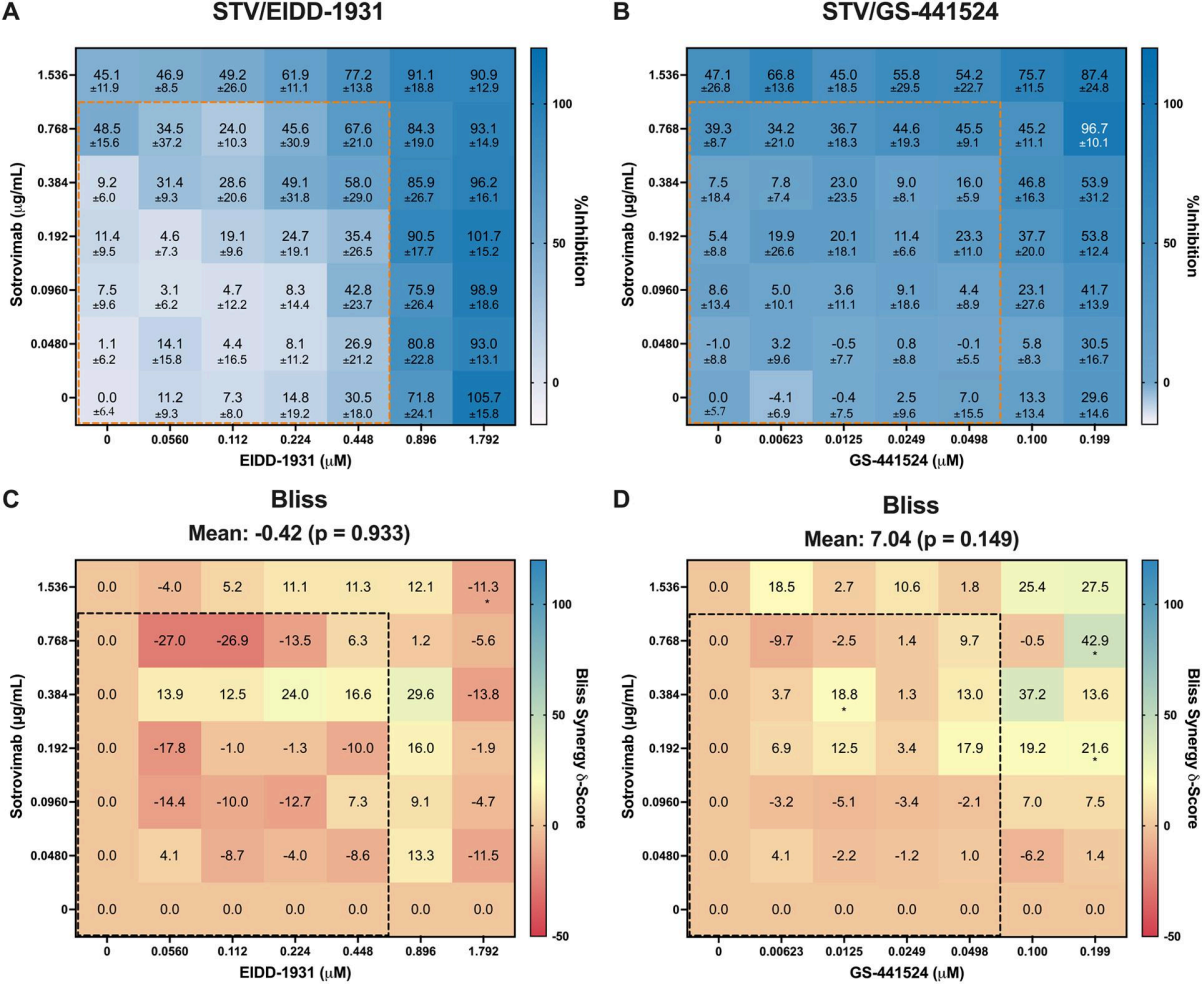
Experimental validation of IDentif.AI-pinpointed STV-based combinations. The interaction map of **(A)** STV/EIDD-1931 and **(B)** STV/GS-441524 combinations in a 7 × 7 checkerboard analysis (*N* = 3). Their %Inhibition data were further analyzed using Bliss independence model, and corresponding synergy scores were used to construct synergy maps for **(C)** STV/EIDD-1931 and **(D)** STV/GS-441524. The dotted boxes indicate the dose ratios within the original L2 concentrations. The average synergy score is provided for each combination assessed by each model. Bliss synergy scores: < −10, between −10 and 10, and > 10 represent antagonistic, additive, and synergistic interactions. Statistical significance of the Bliss synergy scores for each dose ratio is determined using one-sample student *t*-test (*P < 0.05).

STV/EIDD-1931 and STV/GS-441524 combinations were further assessed via a 7 × 7 checkerboard assay (*N* = 3) (Figure 3). Both combinations at different dose ratios were tested in XBB infected Vero E6-ACE2-TMRPSS2 cells. The concentration range of STV started from 2*x* of L2 concentration, while the remaining drugs started at 4*x* of their L2 concentrations with 2-fold serial dilutions. This arrangement accommodated the high potency of STV at L2 concentration (> 60% inhibition), ensuring that drug-drug interactions can be detected (Supplementary Figure S2). Concentration ranges for STV, EIDD-1931, and GS-441524 were 0–1.536 µg/mL, 0–1.792 µM, and 0–0.199 µM, respectively. The %Inhibitions were measured via CPE inhibition assay (Supplementary Data S1). This set of experiments has a Z'-factor of 0.48, suggesting an acceptable assay quality (35, 46). All data were included in the synergy analysis.

**Figure 4:**
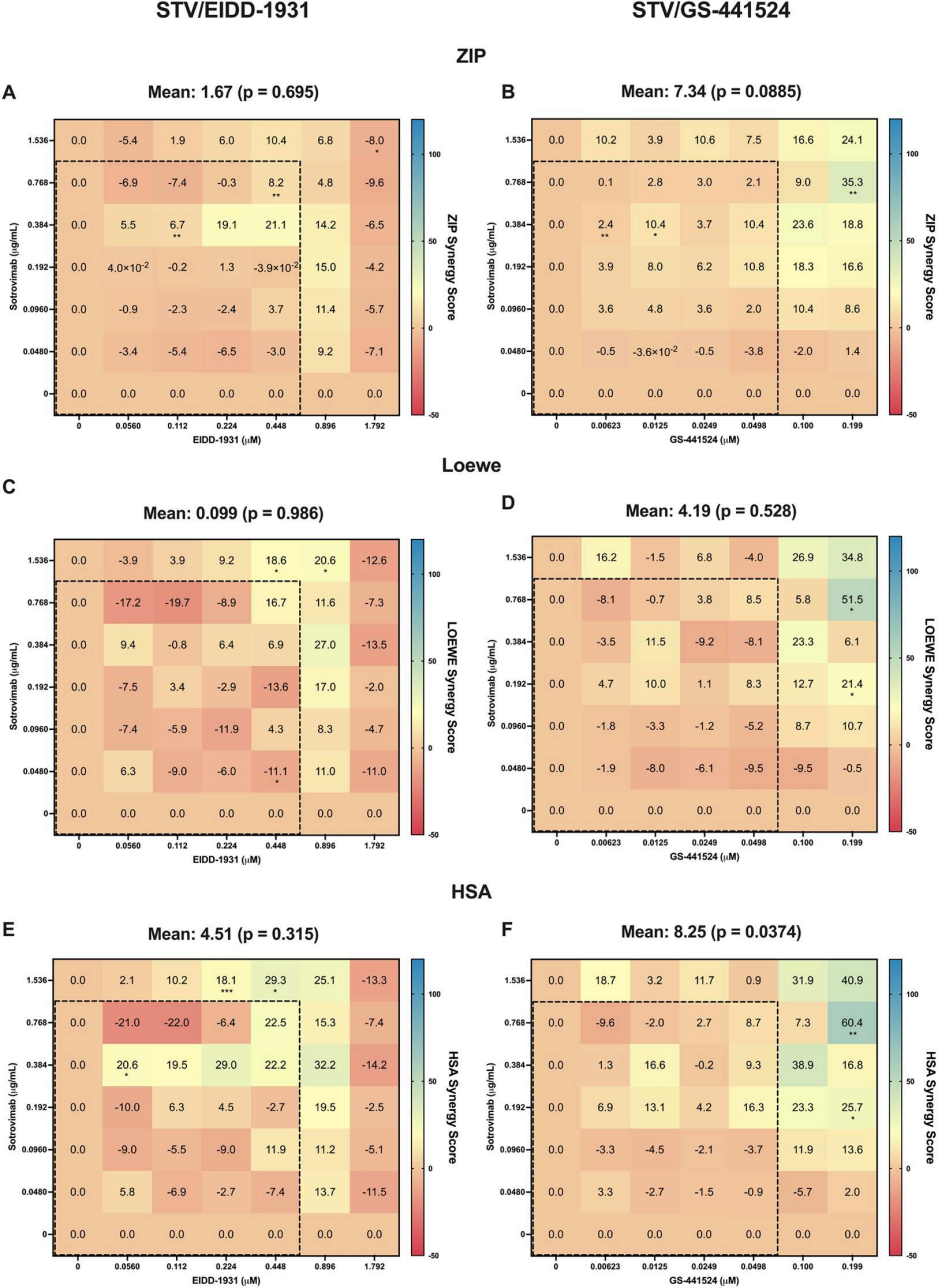
Synergy analysis of %Inhibition data of STV/EIDD-1931 and STV/GS441524 using ZIP, Loewe, and HSA models. The %Inhibition data of each combination were used to analyze drug-drug interactions via **(A,B)** ZIP, **(C,D)** Loewe, and **(E,F)** HSA models, and the resulting synergy scores were used to construct synergy maps for the STV-based combinations. The dotted boxes indicate the dose ratios within the original L2 concentrations. The average synergy score is provided for each combination assessed by each model. Synergy scores: < −10, between −10 and 10, and > 10 represent antagonistic, additive, and synergistic interactions. Statistical significance of the synergy scores for each dose ratio is determined using one-sample student *t*-test (*P < 0.05, **P < 0.01).

In Figure 3A, the interaction map of STV/EIDD-1931 suggested that increasing the concentrations of STV and EIDD-1931 may result in mild interactions that increase the %Inhibition efficacy against the virus. Further assessing the results via the Bliss independence model indicated that STV/EIDD-1931 exhibited dose-dependent synergy (Figure 3C). For instance, even at the highest concentrations for both drugs (STV: 1.536 µg/mL; EIDD-1931: 1.792 µM), the combination did not achieve synergistic interaction. However, when STV was administered at 0.384 µg/mL, the combination resulted in strong synergy. In contrast, antagonistic interactions were observed when STV was administered at 0.768 µg/mL. Additional synergy analysis using ZIP, Loewe, and HSA models similarly revealed the same trends observed from Bliss independence model, suggesting stronger dose-dependent synergy at STV 0.384 µg/mL (Figure 4). Moreover, the dotted box across all interaction and synergy maps represents the dosing range originally assessed by IDentif.AI (0-L2). Notably, IDentif.AI predicted that the interaction of STV/EIDD-1931 at L2 concentrations was mostly additive (Figure 2A). In line with the prediction, Bliss synergy analysis revealed that STV/EIDD-1931 at 0.768 µg/mL (L2) and 0.448 µM (L2), respectively, exhibited additive drug-drug interactions (Synergy score of 6.3) (Figure 3C and Supplementary Data S1 and S2). Similarly, the ZIP model pointed to a statistically significant additive interaction at the dose ratio. However, Loewe and HSA synergy analysis pointed to potential mild synergistic interactions at L2 concentrations for STV/EIDD-1931—0.768 µg/mL (L2) and 0.448 µM (L2), and both models both provided a synergy score of 16.7 and 22.5, respectively (Figure 4). Nonetheless, the interaction of STV/EIDD-1931 is overall dose-dependent, and pinpointing the optimal dose ratio may result in optimal outcomes.

In addition, STV in combination with GS-441524 also similarly demonstrated dose-dependent synergy (Figure 3B). Overall, this combination exhibited stronger synergy than observed in STV/EIDD-1931. For instance, STV and GS-441524 at 0.768 µg/mL and 0.199 µM resulted in monotherapy efficacies of 39.3 ± 8.7%Inhibition and 29.6 ± 14.6%Inhibition, respectively (*N* = 3). However, when paired in combination, STV/GS-441524 achieved 96.7 ± 10.1%Inhibition, pointing to a strong interaction between the drugs (*N* = 3) (Figures 3C, D). Analyzing the dose ratio using Bliss synergy analysis resulted in a statistically significant synergy score of 42.9, further confirming the synergistic drug-drug interaction (Figure 3D and Supplementary Data S2). Critically, the same dose ratio analyzed by ZIP, Loewe, and HSA model also pointed to statistically significant synergistic interactions (Figure 4). Additional dose ratios across the 7 × 7 checkerboard also resulted in strong synergistic interactions analyzed using additional models. The IDentif.AI-predicted interaction of STV/GS-441524 (Figure 2B) also aligned with the analysis in Figures 3B, 3D, and 4. For example, examining the synergy maps of the combination revealed that synergistic interactions were mostly driven by escalating the dose of STV (Figures 3D, 4). In general, STV/GS-441524 exhibited strong synergistic interactions at higher concentrations, especially when GS-441524 was administered at 0.100 and 0.199 µM (Figures 3B, 3D, and 4).

### 3.5 Dose response analysis of STV-based drug combinations

**Figure 5:**
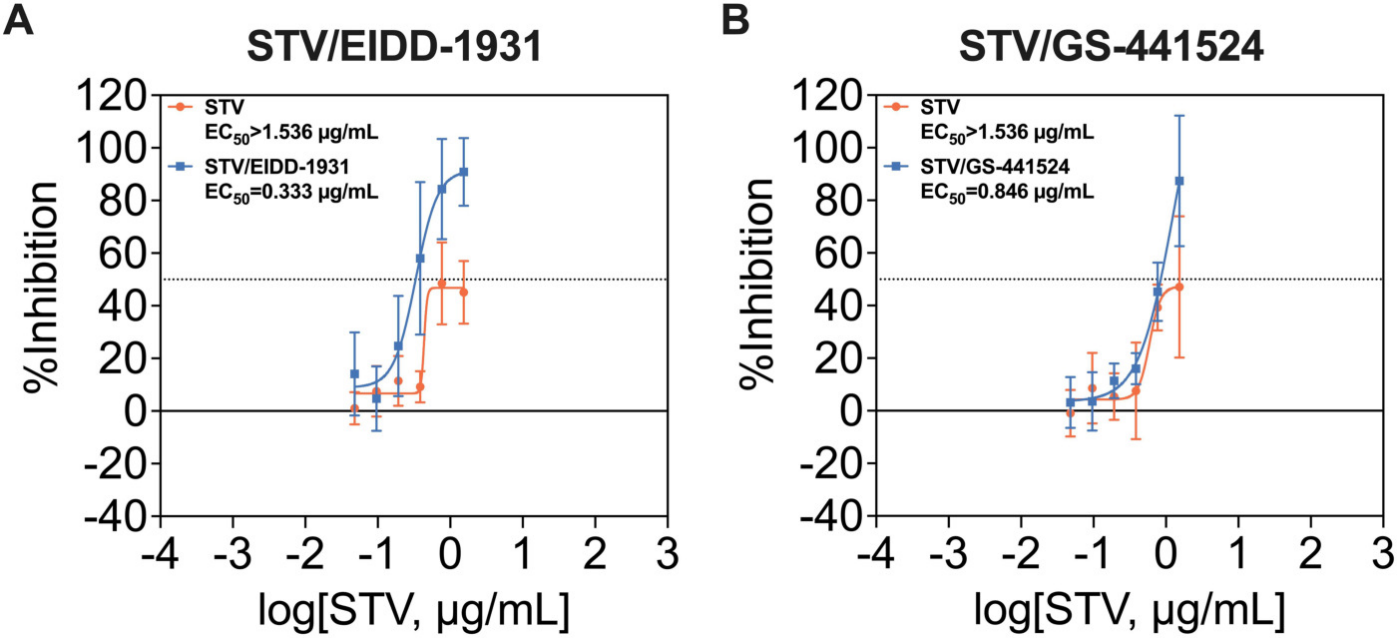
Dose response analysis of **(A)** STV/EIDD-1931 and **(B)** STV/GS-441524 combinations and STV monotherapy (*N* = 3). The %Inhibition values in Figure 3 were used to construct the dose response curves, and the *x*-axis represents the concentrations of STV in µg/mL. The dotted horizontal line corresponds to 50%Inhibition. Sum of squares *F*-test detected statistically significant differences between STV monotherapies and STV-based combinations: **(A)** STV and STV/EIDD-1931 (*P* < 0.0001) and **(B)** STV and STV/GS-441524 *(P* = 0.0179).

Dose response analysis for the STV/EIDD-1931 and STV/GS-441524 combinations was performed by extracting the %Inhibition data from the 7 × 7 checkerboard assays. Figure 5A represents the dose response curve of STV and STV/EIDD-1931. The highest tested STV concentration (1.536 µg/mL) from the 7 × 7 checkerboards did not achieve 50% inhibition and therefore the EC_50_ of STV alone was indicated as >1.536 µg/mL (*N* = 3). However, earlier in the study, the EC_50_ of STV from dose response analysis with a broader range of tested concentrations was 2.449 µg/mL (*N* = 3) (Table 1 and Supplementary Figure S1). In Figure 5A, the dose response curve determined that the EC_50_ of STV/EIDD-1931 was 0.333 µg/mL, indicating that the combination reduced the EC_50_ of STV by 7.4-fold (*N* = 3) (Supplementary Data S1). Similarly, in Figure 5B, the EC_50_ of STV was reduced by 2.9-fold when paired in the STV/GS-441524 combination, which exhibited an EC_50_ of 0.846 µg/mL (*N* = 3) (Supplementary Data S1). Further statistical analysis revealed that there were significant differences between STV and STV-based combinations in the dose response curves, confirming that STV-based combinations can substantially resurrect STV's potency against SARS-CoV-2 (Omicron) XBB variant (Figure 5). In sum, the results indicated that IDentif.AI-pinpointed STV-based combinations were able to achieve synergistic interactions at different dose ratios and importantly, enhance the potency of STV against XBB.

### 3.6 Cytotoxicity assessment of STV-based drug combinations

**Figure 6:**
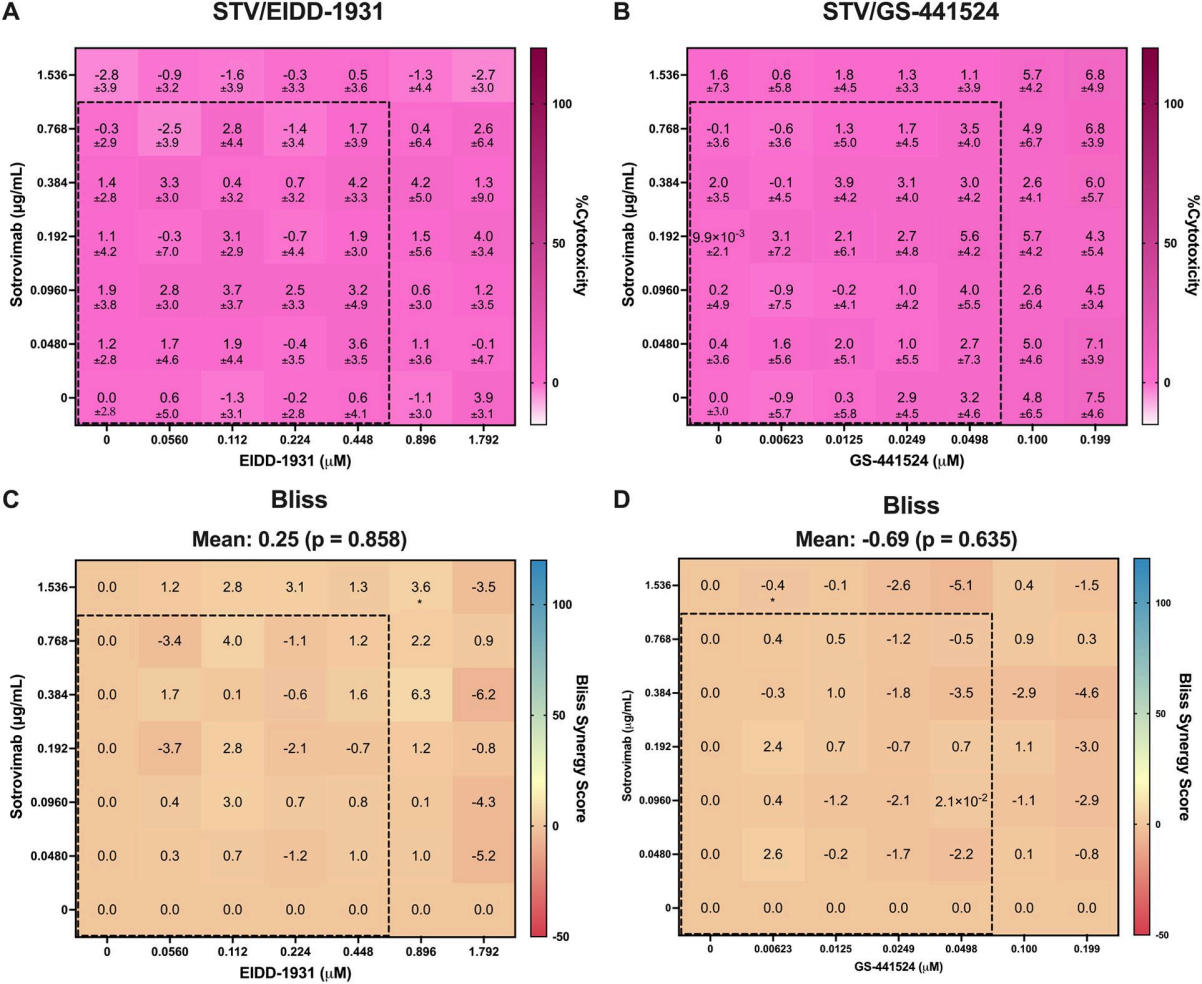
Cytotoxicity analysis of IDentif.AI-pinpointed STV-based combinations. The %Cytotoxicity of **(A)** STV/EIDD-1931 and **(B)** STV/GS-441524 combinations were assessed via a 7 × 7 checkerboard assay (*N* = 3). Their %Cytotoxicity data were further analyzed using Bliss independence model, and corresponding synergy scores were used to construct synergy maps for **(C)** STV/EIDD-1931 and **(D)** STV/GS-441524. The dotted boxes indicate the dose ratios within the original L2 concentrations. The average synergy score is provided for each combination assessed by each model. Bliss synergy scores: < −10, between −10 and 10, and > 10 represent antagonistic, additive, and synergistic interactions. Statistical significance of the Bliss synergy scores for each dose ratio was determined using one-sample student *t*-test (*P < 0.05).

**Figure 7:**
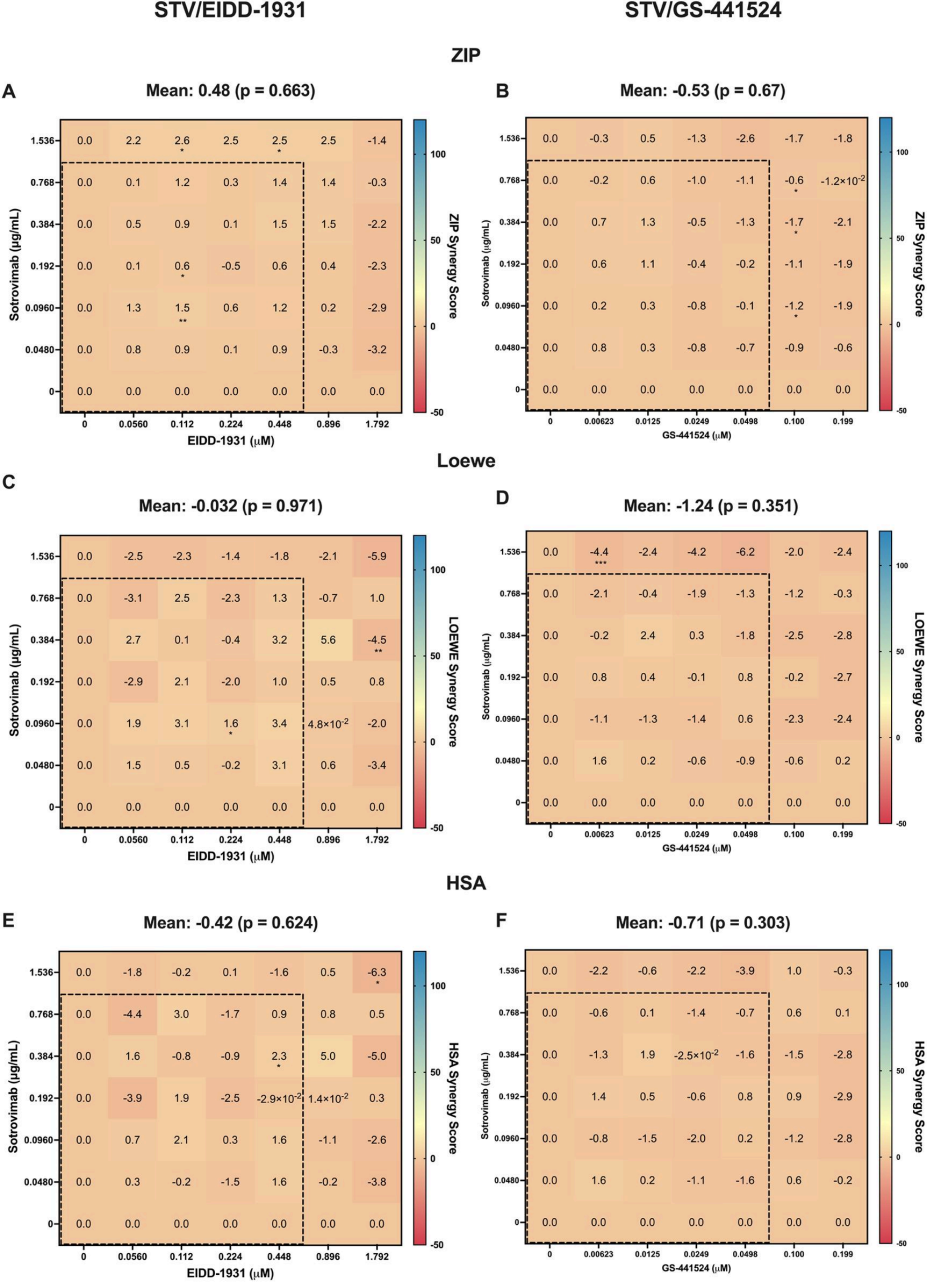
Synergy analysis of %Cytotoxicity data of STV/EIDD-1931 and STV/GS441524 using ZIP, Loewe, and HSA models. The %Cytotoxicity data of each combination were used to analyze drug-drug interactions via **(A,B)** ZIP, **(C,D)** Loewe, and **(E,F)** HSA models, and the resulting synergy scores were used to construct synergy maps for the STV-based combinations. The dotted boxes indicate the dose ratios within the original L2 concentrations. The average synergy score is provided for each combination assessed by each model. Synergy scores: < −10, between −10 and 10, and > 10 represent antagonistic, additive, and synergistic interactions. Statistical significance of the synergy scores for each dose ratio is determined using one-sample student *t*-test (*P < 0.05).

The cytotoxicity of IDentif.AI-pinpointed STV-based combinations was tested in Vero E6-ACE2-TMRPSS2 cells using the same cell viability assay in a 7 × 7 checkerboard design similar to the %Inhibition efficacy (*N* = 3) (Figure 6 and Supplementary Data S1). The %Cytotoxicity of STV/EIDD-1931 and STV/GS-441524 were below 8% showing no apparent toxicity. Even at the highest concentrations, both combinations did not exhibit noticeable %Cytotoxicity. The datasets for both combinations were further analyzed using the Bliss independence, ZIP, Loewe, and HSA models, and no cytotoxicity-induced synergy was observed across all dose ratios for both combinations (Figures 6, 7).

## 4 Discussion

### 4.1 Resurrecting failed therapies through combinatorial strategies

The COVID-19 pandemic has necessitated rapid development and emergency authorization of multiple therapeutics. However, the emergence of SARS-CoV-2 variants substantially changed the susceptibility of various drugs, resulting in the revocation of multiple EUAs. For instance, chloroquine phosphate and hydroxychloroquine sulfate, initially authorized in March 2020 for hospitalized patients, had their EUAs revoked in June 2020 due to limited efficacy and safety concerns (47). Similarly, bamlanivimab lost its standalone treatment EUA in April 2021 as its benefits no longer outweighed the risks against emerging variants (47, 48), and REGEN-COV (casirivimab and imdevimab) became ineffective against the Omicron variants, resulting in EUA revocation in January 2022 (48). These revocations highlight the challenges associated with maintaining durable therapeutic efficacy during rapid viral evolution and the need for dynamic pandemic preparedness strategies capable of responding to emerging variants.

In this study, IDentif.AI was harnessed to address the substantial reduction in STV potency against emerging SARS-CoV-2 variants, particularly the XBB variant. STV was initially granted FDA EUA in May 2021 for the treatment of mild-to-moderate COVID-19 patients (30). In early 2022, more than 50% of infections were caused by the BA.2 Omicron sub-variant. As a result, STV's activity against the virus substantially reduced, leading to restrictions on the use of STV for COVID-19 treatment. In April 2022, STV was no longer authorized to treat COVID-19 until further notice in the United States (49). Similarly, the National Centre for Infectious Diseases (Singapore) also no longer recommended the use of STV for COVID-19 treatment (36).

To resurrect the potency of STV, IDentif.AI interrogated the drug-drug interaction space comprising of STV and five additional drug candidates including antivirals and an anti-inflammatory Janus kinase inhibitor (JAK1/2—BRT). Notably, IDentif.AI-prioritized STV/EIDD-1931 and STV/GS-441524 combinations substantially reduced the EC_50_ of STV by up to 7-fold (Figure 5). These findings suggest that pairing MABs with antivirals may potentially compensate for the reduced neutralization potency from mutations in SARS-CoV-2 spike protein. Furthermore, while STV targets the spike protein, EIDD-1931 and GS-441524 both inhibit viral replications, potentially enabling complementary approaches against rapid emergence of variants.

The findings of this study are further supported by recent work from Fiaschi et al., which demonstrated that pairing STV with other drugs may restore the potency of STV against SARS-CoV-2 variants. Notably, their study also validated the STV/RDV combination, which was among the top STV-based combinations pinpointed by IDentif.AI (Supplementary Table S3), and reported a 4-fold reduction in STV EC_50_ (50). Importantly, GS-441524, which was prioritized and validated in combination with STV, is the parent nucleoside of RDV. Collectively, these studies demonstrated the combination therapies may help extend the clinical relevance and potentially “resurrect” therapeutics that have become less effective due to virus's evolution, resistance, and other reasons leading to reduced efficacy.

Although IDentif.AI-prioritized STV-based drug combinations reduced STV EC_50_ by up to 7-fold, the clinical implications of the observed “resurrection” effect should be carefully interpreted. For example, Omicron spike proteins have been reported to exhibit 26 to 34-fold greater resistance to vaccine-elicited antibodies compared to earlier SARS-CoV-2 variants (51). Moreover, Omicron variant has been associated with substantially higher transmissibility compared with the Delta variant (52). Therefore, the clinical translatability of these observations in preclinical models as well as humans should be further investigated, especially whether these enhancements are sufficient to “resurrect” STV efficacy for downstream clinical actionability. Nonetheless, this study highlights the potential of IDentif.AI to rapidly identify potential drug-drug interactions that may restore or even expand the clinical relevance of therapeutics.

### 4.2 Dose-dependent synergistic interactions

In this study, IDentif.AI-prioritized STV-based drug combinations were further evaluated using 7 × 7 checkerboard assays and subsequently, analyzed using Bliss, ZIP, HSA, and Loewe synergy models. Notably, the interactions of STV and EIDD-1931 at L2 concentrations were evaluated as mostly additive by Bliss and ZIP models, while the Loewe and HSA models pointed to mildly synergistic for the same dose ratio (Figures 3, 4). The differences in drug-drug interaction analysis may arise from distinct pharmacological and mathematical assumptions of each model (33). For example, the HSA model assumes that the expected efficacy of a combination is equivalent to the maximal efficacy of either drug alone (33). In contrast, the Bliss independence model assumes that two drugs interact independently through different mechanisms. Nonetheless, evaluating drug combinations using multiple synergy frameworks may provide a more comprehensive understanding of drug-drug interactions under various pharmacological and mathematical assumptions.

Notably, the Bliss, ZIP, Loewe, and HSA models consistently point to a statistically significant synergistic interaction for STV/GS-441524 at 0.768 µg/mL and 0.199 µM, respectively (Figures 3, 4). This observation further confirmed the identified STV/GS-441524 synergistic interaction at this specific dose ratio as confirmed by multiple synergy frameworks. Moreover, even though dose-dependent synergistic interactions were identified across multiple dose ratios, the average synergy scores across the entire 7 × 7 checkerboard for both STV-based combinations were between −10 and 10, indicating overall additive drug-drug interactions (Figures 3, 4). Notably, certain dose ratios within the checkerboards also exhibited antagonistic interactions, where reduced %Inhibition efficacy was observed when drugs interacted. Together, these findings further reinforce a central principle of combinatorial strategies: therapeutic efficacy is not only dependent on pairing the correct drugs in combinations, but also identifying their optimal dose ratios (12–14, 16, 19, 21–26, 53).

The clinical relevance and actionability of the identified synergistic dose ratios for STV-based drug combinations should also be carefully interpreted. First, the optimal dose ratios identified *in vitro* using Vero E6 cells may not directly translate to preclinical models or humans due to differences in multiple factors, including host immune response, pharmacokinetics, and more. Similarly, synergistic interactions may also vary across biological systems as well as individual patients due to inter- and intra-patient variability (21, 23, 25, 53). As a result, the optimal synergistic dose ratios identified in this study may require further re-optimization during downstream preclinical and clinical validation. In summary, the findings of this study further emphasize the importance of optimizing drug combinations and their dose ratios against continually evolving viruses.

### 4.3 Prioritizing accessible STV-based drug combinations

Of all the IDentif.AI-pinpointed STV-based drug combinations, STV/EIDD-1931 and STV/GS-441524 were prioritized not only for therapeutic compliance, but also accessibility. Intravenously administered drugs such as RDV typically require frequent clinical visits accompanied by intravenous injection systems, which may reduce therapeutic compliance and treatment accessibility (44). For example, RDV may not be broadly accessible to non-hospitalized patients. Therefore, pairing STV with orally available antivirals was prioritized in this study to improve accessibility and practicality of combination therapies during the pandemic (43, 44). Even though STV itself is administered intravenously, combining it with orally available drugs may still improve overall accessibility, adherence, and practicality compared with combinations comprised of multiple intravenously administered drugs.

Furthermore, EIDD-1931 is the active metabolite of molnupiravir, an orally administered antiviral that received EUA for the treatment of mild-to-moderate COVID-19 in December 2021. Similarly, multiple orally available antivirals including GS-621763 and VV116 are derived from GS-441524 (54), which is the parent nucleoside of RDV. Notably, a multi-center phase III randomized controlled trial reported that VV116 reduced the time to achieve sustained clinical symptom resolution in COVID-19 patients (55). Together, considering the clinical practicality and accessibility of these antivirals, STV/EIDD-1931 and STV/GS-441524 were prioritized in this study for additional synergy analyses to evaluate their drug-drug interactions (Figures 3–5). Beyond enhancing STV potency through combinatorial designs, prioritizing fully or partially orally available regimens may offer some advantages in the context of pandemic readiness: reducing hospitalization burden and alleviating pressure on healthcare systems. In sum, these STV-based combinations with orally available antivirals may provide practical advantages for clinical implementation, especially during outbreak and emergency scenarios.

In this study, IDentif.AI interrogated the drug-drug interaction space using 50 OACD combinations and pinpointed STV-based drug combinations that substantially reduced STV EC_50_ against SARS-CoV-2 Omicron XBB *in vitro*. Beyond IDentif.AI, alternative AI-enabled approaches, such as high throughput screening, may also be harnessed to restore the potency of previously failed or ineffective therapeutics (56–58). Importantly, assessing the differences and complementary advantages of IDentif.AI with other existing/emerging AI-driven approaches may help establish frameworks that improve existing drug development workflows for future pandemic readiness.

### 4.4 Integration of complementary AI-based optimization approaches

Aside from IDentif.AI, multiple AI-driven approaches have also contributed to the optimization of drug combinations for infectious diseases. For example, Wong et al. proposed a metamodel antimicrobial cocktail optimization (MACO) framework consisting of factorial regression and response surface regression to facilitate combinatorial optimization. Specifically, the framework only requires 18 trials to derive synergistic drug cocktails against bacteria (7). In another study, supervised machine learning was employed to predict synergy between antimicrobial peptides and agents by leveraging the Database of Antimicrobial Activity and Structure of Peptides (DBAASP) and DrugBank (59). This approach was able to achieve accuracy up to 76.92%. Moreover, a stochastic search algorithm was utilized to determine effective drug combinations against vesicular stomatitis virus, while substantially reducing the amount of trials needed for optimization (6). Importantly, IDentif.AI differs from these approaches in that the platform only requires small data-driven prospective interrogation of drug-drug interaction space with a single optimization step, rather than iteratively steps toward optimal combinatorial designs. Such approach may present notable advantages during pandemics: the ability to determine optimal combinations with small datasets that can be obtained in one single iteration.

Although the above-mentioned AI-driven optimization approaches offer distinct solutions for drug combination optimization, they may provide complementary advantages that further improve optimization workflows for future pandemic readiness. Future integration of AI-enabled and computational approaches including IDentif.AI, machine learning models, AI-driven high throughput screening, and stochastic optimization frameworks may together improve drug development workflows for emerging infectious diseases. For example, predictive AI models may be harnessed to prioritize actionable drug candidates prior to combinatorial optimization. Subsequently, IDentif.AI and other AI-driven optimization platforms may pinpoint actionable, efficacious drug combinations. The integration of complementary AI-enabled and computational approaches may help establish relevant, adaptive and scalable framework that strengthens future pandemic readiness against emerging pathogens.

### 4.5 Study limitations

The IDentif.AI platform presented a strategic approach to potentially restore the efficacy of STV against emerging SARS-CoV-2 variants. However, several study limitations should be considered when interpreting the findings in this study. First, IDentif.AI-prioritized STV/EIDD-1931and STV/GS-441524 combinations were optimized from a pool of only 6 drug candidates. Expanding the drug library to include a broader range of drug classes may result in other STV-based combinations capable of enhancing STV efficacy against XBB. Additionally, IDentif.AI only assessed three concentrations levels (L0, L1, and L2) for each of the 6 drug candidates. As observed in the synergy analysis, drug combinations may exhibit dose-dependent interactions at higher or lower concentrations. Thus, when assessing only three concentrations levels, potential drug-drug interactions at lower or higher concentrations may not be detected by IDentif.AI. In future experiments, alternative OACD designs incorporating additional concentrations levels may potentially detect a broader range of drug-drug interactions. Furthermore, all IDentif.AI experiments were conducted using SARS-CoV-2 Omicron XBB subvariants. Thus, the prioritized combinations may not exhibit similar efficacy against other variants or subvariants (e.g., BQ.1). Lastly, to develop drug combinations with broader applicability, the IDentif.AI workflows should be conducted across multiple variants. From these assessments, the platform may identify broadly applicable combinatorial designs that are effective against multiple variants.

Furthermore, the experimental model used in this study also has several limitations. As discussed in previous sections, the IDentif.AI-prioritized combinations were only validated *in vitro*, and the enhanced efficacy of STV requires further downstream validation in preclinical models and clinical trials. Moreover, while the selected L1/L2 drug concentrations were intended to reflect clinically achievable drug exposures, IDentif.AI *in vitro* experiments may not fully recapitulate human pharmacokinetics, including plasma protein binding (e.g., free drug levels) and tissue distribution. In addition, Vero E6-ACE2-TMRPSS2 cells may not fully represent the primary site of entry in the respiratory tract, including the host immune responses (60). Furthermore, the %Inhibition efficacy of this study was measured through virus-induced CPE. Although CPE assays provide robust screening of antiviral activity, additional validation in other experimental assays may provide further insights into the clinical relevance of IDentif.AI-prioritized combinations.

Importantly, RDV and NMV are known substrates of P-glycoprotein (P-gp), or Multi-drug Resistance Protein 1 (MDR1), which is highly expressed in Vero E6 cells (61, 62). Although Vero E6 cells are particularly relevant for antiviral drug research, the efflux activity of P-gp may substantially impact the *in vitro* efficacy of therapeutics such as NVM and RDV, which may have been affected by this experimental model. Future studies may address this limitation through co-incubation with P-gp inhibitors to account for the efflux activity. Alternatively, optimizing drug combinations in other cell lines like A549 (human lung adenocarcinoma) cells or primary bronchial epithelial cells may provide further insights into top performing drug combinations without the efflux activity of P-gp. Recently, Zhu et al. harnessed CRISPR-Cas9 technology to generate a P-gp gene knockout Vero E6 cell line (VeroE6-Pgp-KO) (62), which may provide a potential solution for *in vitro* screening of drugs that are P-gp substrates. Addressing these limitations pertaining to experimental model may lead to substantially different drug-drug interactions.

In the dose response analysis stage of this study, EC_10_ and EC_20_ values were extracted and subsequently used to define the L1 and L2 concentrations for selected drugs (Table 1 and Supplementary Figure S1). However, experimental validation of the STV L2 concentration, which was based on EC_20_, resulted in substantially higher %Inhibition (> 60%) (Supplementary Figure S2). Repeated experiments also resulted in substantial variation in the potency of STV, potentially reflecting the sensitive response of the cells/virus to STV treatment. Furthermore, after completion of the study, updated pharmacological and pharmacokinetic data of obeldesivir (GS-443902), an oral prodrug of nucleoside GS-441524, were published (63). The L1 and L2 concentrations for GS-441524 were based on earlier C_max_ value available at the time of experimental design (Table 1).

The COVID-19 pandemic has highlighted the need for adaptable therapeutic strategies in the face of emerging SARS-CoV-2 variants as well as other pathogens (e.g., AMR). As mutations render certain treatments less effective, it is crucial to develop methods for rapidly identifying and optimizing drug combinations. This study demonstrates the potential of AI-enabled approaches, such as IDentif.AI, to revitalize existing therapeutics like STV against emerging variants. By identifying synergistic combinations of STV with EIDD-1931 and GS-441524, a significant 7-fold reduction in STV EC_50_ was achieved. This approach offers a promising pathway for rapidly repurposing and enhancing the efficacy of existing or previously ineffective drugs against evolving viral threats, potentially providing a valuable tool in the ongoing battle against COVID-19 and future pandemics.

## Data Availability

The original contributions presented in the study are included in the article/Supplementary Material, further inquiries can be directed to the corresponding author.
