# Supplementary material for: Optimizing drug combinations to resurrect the potency of failed antibody therapy against emerging COVID-19 variants using IDentif.AI
Source: Front Digit Health. 2026 Jun 2;8:1744623. doi: 10.3389/fdgth.2026.1744623 (PMC13269110; doi:10.3389/fdgth.2026.1744623)
Supplement: Supplementary file 3 [file Datasheet3.docx]

Supplementary Material

# Supplementary Data

**Supplemental Data 1.** The raw %Inhibition and %Cytotoxicity data for dose response curves, IDentif.AI analysis, and validation checkerboards are listed according to their respective drug concentrations. The synergy scores for checkerboard assays are also summarized in the Excel sheet.

**Supplemental Data 2.** IDentif.AI-pinpointed drug combinations containing four drugs or less are summarized in the Excel sheet. The combinations are listed with their corresponding IDentif.AI-predicted %Inhibition values and rank within the drug interaction space comprising of 729 combinations.

# Supplementary Figures and Tables

## Supplementary Tables

**Table S1.** **A resolution VI 50-combination orthogonal array composite design (OACD) for 6-drug optimization.** -1, 0, and 1 represent L0, L1, and L2 concentrations, respectively. Abbreviations: nirmatrelvir (NMV), remdesivir (RDV), baricitinib (BRT), and sotrovimab (STV).

|  | **NMV** | **RDV** | **GS-441524** | **BRT** | **STV** | **EIDD-1931** |
| --- | --- | --- | --- | --- | --- | --- |
| 1 | -1 | -1 | -1 | -1 | -1 | -1 |
| 2 | 1 | -1 | -1 | -1 | -1 | 1 |
| 3 | -1 | 1 | -1 | -1 | -1 | 1 |
| 4 | 1 | 1 | -1 | -1 | -1 | -1 |
| 5 | -1 | -1 | 1 | -1 | -1 | 1 |
| 6 | 1 | -1 | 1 | -1 | -1 | -1 |
| 7 | -1 | 1 | 1 | -1 | -1 | -1 |
| 8 | 1 | 1 | 1 | -1 | -1 | 1 |
| 9 | -1 | -1 | -1 | 1 | -1 | 1 |
| 10 | 1 | -1 | -1 | 1 | -1 | -1 |
| 11 | -1 | 1 | -1 | 1 | -1 | -1 |
| 12 | 1 | 1 | -1 | 1 | -1 | 1 |
| 13 | -1 | -1 | 1 | 1 | -1 | -1 |
| 14 | 1 | -1 | 1 | 1 | -1 | 1 |
| 15 | -1 | 1 | 1 | 1 | -1 | 1 |
| 16 | 1 | 1 | 1 | 1 | -1 | -1 |
| 17 | -1 | -1 | -1 | -1 | 1 | 1 |
| 18 | 1 | -1 | -1 | -1 | 1 | -1 |
| 19 | -1 | 1 | -1 | -1 | 1 | -1 |
| 20 | 1 | 1 | -1 | -1 | 1 | 1 |
| 21 | -1 | -1 | 1 | -1 | 1 | -1 |
| 22 | 1 | -1 | 1 | -1 | 1 | 1 |
| 23 | -1 | 1 | 1 | -1 | 1 | 1 |
| 24 | 1 | 1 | 1 | -1 | 1 | -1 |
| 25 | -1 | -1 | -1 | 1 | 1 | -1 |
| 26 | 1 | -1 | -1 | 1 | 1 | 1 |
| 27 | -1 | 1 | -1 | 1 | 1 | 1 |
| 28 | 1 | 1 | -1 | 1 | 1 | -1 |
| 29 | -1 | -1 | 1 | 1 | 1 | 1 |
| 30 | 1 | -1 | 1 | 1 | 1 | -1 |
| 31 | -1 | 1 | 1 | 1 | 1 | -1 |
| 32 | 1 | 1 | 1 | 1 | 1 | 1 |
| 33 | -1 | -1 | -1 | -1 | -1 | -1 |
| 34 | -1 | 0 | 0 | 0 | 0 | 0 |
| 35 | -1 | 1 | 1 | 1 | 1 | 1 |
| 36 | 0 | -1 | -1 | 0 | 0 | 1 |
| 37 | 0 | 0 | 0 | 1 | 1 | -1 |
| 38 | 0 | 1 | 1 | -1 | -1 | 0 |
| 39 | 1 | -1 | 0 | -1 | 1 | 0 |
| 40 | 1 | 0 | 1 | 0 | -1 | 1 |
| 41 | 1 | 1 | -1 | 1 | 0 | -1 |
| 42 | -1 | -1 | 1 | 1 | 0 | 0 |
| 43 | -1 | 0 | -1 | -1 | 1 | 1 |
| 44 | -1 | 1 | 0 | 0 | -1 | -1 |
| 45 | 0 | -1 | 0 | 1 | -1 | 1 |
| 46 | 0 | 0 | 1 | -1 | 0 | -1 |
| 47 | 0 | 1 | -1 | 0 | 1 | 0 |
| 48 | 1 | -1 | 1 | 0 | 1 | -1 |
| 49 | 1 | 0 | -1 | 1 | -1 | 0 |
| 50 | 1 | 1 | 0 | -1 | 0 | 1 |

**Table S2.** IDentif.AI-estimated coefficients of the second order quadratic series used to predict all possible combinations in the parameter space consisting of 6 drugs at three concentration levels. Abbreviations: nirmatrelvir (NMV), remdesivir (RDV), baricitinib (BRT), sotrovimab (STV), and squared error (SE).

| **Term** | **Estimate** | **SE** | ***t*-statistic** | ***p*-value** |
| --- | --- | --- | --- | --- |
| Intercept | 74.327 | 5.702 | 13.035 | 1.70 × 10^-27^ |
| NMV | 0.591 | 1.673 | 0.353 | 0.72 |
| RDV | 5.742 | 1.664 | 3.451 | 7.01 × 10^-4^ |
| GS-441524 | 5.239 | 1.663 | 3.151 | 1.92 × 10^-3^ |
| BRT | -7.344 | 1.682 | -4.366 | 2.18 × 10^-5^ |
| STV | 23.267 | 1.666 | 13.970 | 3.52 × 10^-30^ |
| EIDD-1931 | 16.643 | 1.667 | 9.985 | 7.94 × 10^-19^ |
| NMV:GS-441524 | -3.308 | 1.766 | -1.873 | 0.06 |
| RDV:EIDD-1931 | -4.337 | 1.737 | -2.497 | 0.01 |
| BRT:STV | -3.609 | 1.738 | -2.077 | 0.04 |
| STV:EIDD-1931 | -4.701 | 1.763 | -2.667 | 8.39 × 10^-3^ |
| GS-441524^2^ | -9.563 | 4.876 | -1.961 | 0.05 |
| BRT^2^ | -8.738 | 4.920 | -1.776 | 0.08 |
| **Model Statistics** | | | | |
| Number of Observations | | | 186 | |
| Error Degrees of Freedom | | | 173 | |
| Root Mean Squared Error | | | 20.5 | |
| R^2^ | | | 0.729 | |
| Adjusted R^2^ | | | 0.71 | |
| *F*-statistic vs. Constant Model | | | 38.8 | |
| *p*-value | | | 8.45 × 10^-43^ | |
| Fitting Correlation | | | 0.854 | |

**Table S3. IDentif.AI-pinpointed top 2-drug combinations.** The concentration levels of the drugs are included in the parenthesis: (1) and (2) represent L1 and L2 concentrations, respectively. The IDentif.AI predicted %Inhibitions are also included for each combination. Abbreviations: sotrovimab (STV), remdesivir (RDV), and nirmatrelvir (NMV).

| **Rank (Out of 729)** | **Drug 1** | **Drug 2** | **Predicted %Inhibition** |
| --- | --- | --- | --- |
| 96 | STV (2) | EIDD-1931 (2) | 91.64 |
| 199 | STV (2) | RDV (2) | 79.24 |
| 216 | STV (2) | GS-441524 (1) | 77.20 |
| 227 | STV (2) | GS-441524 (2) | 76.18 |
| 237 | STV (2) | EIDD-1931 (1) | 75.37 |
| 303 | STV (1) | EIDD-1931 (2) | 69.47 |
| 309 | STV (2) | RDV (1) | 69.16 |
| 336 | STV (2) | NMV (2) | 66.88 |
| 352 | EIDD-1931 (2) | GS-441524 (1) | 65.40 |
| 364 | EIDD-1931 (2) | GS-441524 (2) | 64.39 |

**Table S4. IDentif.AI-pinpointed top 3-drug and 4-drug combinations.** The concentration levels of the drugs are included in the parenthesis: (1) and (2) represent L1 and L2 concentrations, respectively. The IDentif.AI predicted %Inhibitions are also included for each combination. Abbreviations: sotrovimab (STV), remdesivir (RDV), and nirmatrelvir (NMV).

| **Rank** | **Drug 1** | **Drug 2** | **Drug 3** | **Drug 4** | **Predicted %Inhibition** |
| --- | --- | --- | --- | --- | --- |
| **Top 3-drug Combinations** | | | | | |
| 15 | STV (2) | EIDD-1931 (2) | GS-441524 (1) |  | 109.75 |
| 20 | STV (2) | EIDD-1931 (2) | GS-441524 (2) |  | 108.74 |
| 51 | STV (2) | EIDD-1931 (2) | NMV (2) |  | 99.44 |
| 62 | STV (2) | RDV (2) | GS-441524 (1) |  | 97.35 |
| 66 | STV (2) | RDV (2) | GS-441524 (2) |  | 96.34 |
| **Top 4-drug Combinations** | | | | | |
| 3 | STV (2) | EIDD-1931 (2) | GS-441524 (1) | RDV (2) | 112.56 |
| 6 | STV (2) | EIDD-1931 (2) | GS-441524 (2) | RDV (2) | 111.55 |
| 8 | STV (2) | EIDD-1931 (2) | GS-441524 (1) | RDV (1) | 111.16 |
| 10 | STV (2) | EIDD-1931 (2) | GS-441524 (1) | NMV (2) | 110.94 |
| 12 | STV (2) | EIDD-1931 (2) | GS-441524 (1) | NMV (1) | 110.35 |

## Supplementary Figures

**Figure S1. Dose response analysis of the 6 selected drug candidates.** Each of the drugs was exposed to Vero E6-ACE2-TMPRSS2 cells infected with live virus at 12 different concentrations. Drug treatments were incubated for 72 h and their virus-induced CPE inhibition and cytotoxicity effects were measured (N = 3). The horizontal line corresponds to 50 %Inhibition. The dotted circles in EIDD-1931 represent outliers that were removed from the dose response analysis due to high cytotoxicity (> 15%). Abbreviations: nirmatrelvir (NMV), remdesivir (RDV), baricitinib (BRT), and sotrovimab (STV).


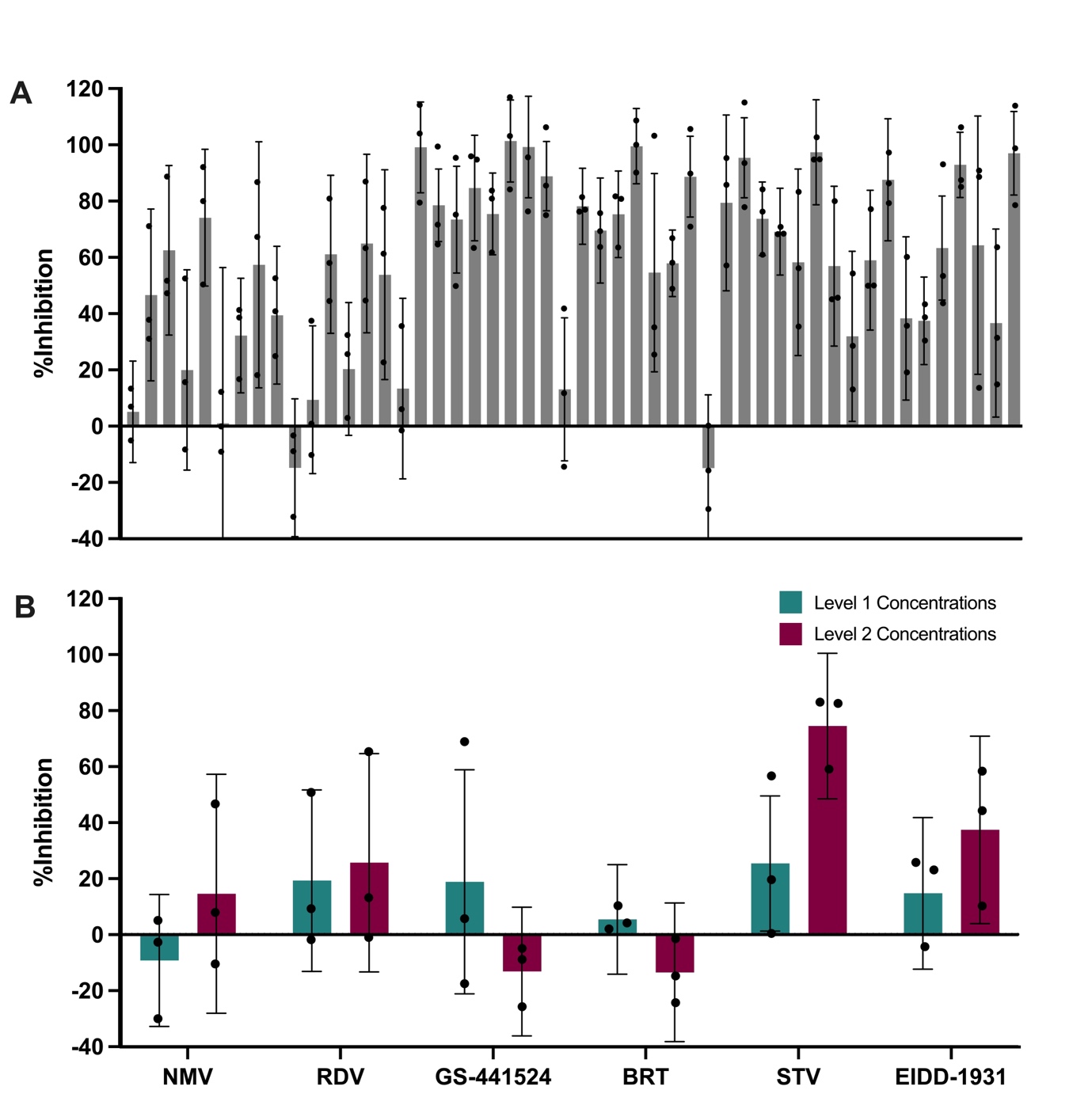


**Figure S2. Monotherapy and combinatorial assessment of the selected drugs.** Prospectively obtained %Inhibition for (A) 50 OACD combinations and (B) each drug at L1 and L2 concentrations (N = 3). Abbreviations: nirmatrelvir (NMV), remdesivir (RDV), baricitinib (BRT), and sotrovimab (STV).

**Figure S3. Residual-based outlier analysis on the %Inhibition data.** No outlier was identified and all data were included in the IDentif.AI analysis (N = 3).
